# Differences in Antioxidant and Lipid Handling Protein Expression Influence How Cells Expressing Distinct Mutant TP53 Subtypes Maintain Iron Homeostasis

**DOI:** 10.3390/cells11132064

**Published:** 2022-06-29

**Authors:** Cameron J. Cardona, Evan R. Hermann, Kate N. Kouplen, Steven D. Hartson, McKale R. Montgomery

**Affiliations:** 1 Department of Nutritional Sciences, Oklahoma State University, Stillwater, OK 74078, USA; cameron.cardona@okstate.edu (C.J.C.); evanhermann93@gmail.com (E.R.H.); 2 Department of Integrative Biology, Oklahoma State University, Stillwater, OK 74078, USA; kkouple@okstate.edu; 3 Biochemistry and Molecular Biology, Oklahoma State University, Stillwater, OK 74078, USA; steven.hartson@okstate.edu

**Keywords:** mutant TP53, iron metabolism, ferroptosis, cancer, proteomics

## Abstract

The tumor suppressor TP53 is the most commonly mutated gene in human cancers, and iron is necessary for cancer cell growth and proliferation, but there is a significant gap in knowledge for how the two cooperate to affect cellular physiology. Elucidating this role is complicated, however, because each TP53 mutation subtype exhibits unique phenotypic responses to changes in iron availability. The goal of this work was to determine how cells expressing distinct TP53 mutation subtypes respond to iron restriction. Utilizing a reverse genetics approach, we generated eight isogenic cell lines that either lacked TP53 expression, expressed wild-type TP53, or expressed one of the six most common TP53 “hotspot” mutations. We then employed isobaric peptide labeling and mass spectrometry to quantitively measure changes in global protein expression, both in response to induction of mutant TP53 expression, and in response to iron chelation. Our findings indicate that mutant TP53-dependent sensitivities to iron restriction are not driven by differences in responsiveness to iron chelation, but more so by mutant TP53-dependent differences in cellular antioxidant and lipid handling protein expression. These findings reinforce the importance of distinguishing between TP53 mutation subtypes when investigating approaches to target mutant TP53. We also identify unique TP53-dependent perturbances in protein expression patterns that could be exploited to improve iron-targeted chemotherapeutic strategies.

## 1. Introduction

The essential yet toxic nature of iron is largely attributed to its redox potential and its propensity to form free radicals, respectively. Cancer cells use extravagant amounts of iron to sustain their high rates of proliferation and growth, but the higher amounts of labile iron within cancer cells increases their susceptibility to oxidative stress and programmed cell death pathways such as ferroptosis [1,2]. Thus, ferroptosis activation is an attractive anticancer approach.

The tumor suppressor gene TP53 is an important mediator of ferroptosis in human cancer cells, but contradictory roles have been reported [3,4,5,6]. These mixed findings may be attributable to the context-dependent upregulation of canonical TP53 targets, which appears to be critical for suppression of ferroptosis [4]. However, the numerous and divergent TP53 mutation phenotypes likely also contribute to these confounding results. As TP53 is mutated in more than half of all human cancers, there is a critical need to understand how distinct TP53 mutation types influence cancer outcomes.

Irrespective of the TP53 mutation type, one common therapeutically targetable TP53 mutation outcome may be increased sensitivity to ferroptosis [4,7,8]. However, the mechanisms contributing to augmented ferroptosis sensitivity in mutant TP53-expressing cells are not clear. For example, despite similar reductions in cell viability in response to ferroptosis induction, cells expressing distinct TP53 mutations display dissimilar changes in iron regulatory protein activity and iron-related gene expression [9]. Moreover, the regulation of iron homeostasis in response to changes in iron availability appears to be unique to individual TP53 mutation types. The goal of this study was to determine how cells expressing distinct TP53 mutations maintain iron homeostasis in response to reduced iron availability. We also examined how individual TP53 mutations themselves can influence sensitivity to changes in cellular iron levels and augment ferroptosis sensitivity.

## 2. Materials and Methods

### 2.1. Cell Line Construction and Care

TP53 null, H1299 cells, were purchased from the American Type Culture Collection (ATCC; Manassas, VA, USA) and maintained in 10-1040-CV RPMI 1640 with L-glutamine (Corning; Corning, NY, USA) containing 10% tetracycline-free FBS (Atlanta Biologicals, Norcross, GA, USA), 100 IU/mL penicillin, and 100 µg/mL streptomycin (Corning; Corning, NY, USA). Isogenic cell lines expressing pcDNA6/TR and an empty pcDNA5/TO plasmid (H1299), wild-type TP53 gene, or one of the representative TP53 hotspot mutations, were generated by transfection with lipofectamine 3000 (Thermo Fisher Scientific, Waltham, MA, USA) in serum-reduced media, followed by antibiotic selection in complete media, and validation as previously described [8]. All cell lines were maintained in the media described above supplemented with 600 µg/mL hygromycin B and 10 µg/mL blasticidin. TP53 expression was induced by treatment with 10 µg/mL tetracycline. Cells expressing endogenous WT TP53 (SW48) or an endogenous R273H (MDA-MB-468), R248Q (HCC70), R282G (NCI-H510), R175H (AU565), G245S (SU.86.86), or R249S (BT549) TP53 mutation were also obtained from ATCC and were maintained according to the instructions provided on the provider’s website. All cells were kept in a humidity and temperature-controlled cell incubator set to 5% CO_2_, 95% humidity, and 37 °C.

### 2.2. Treatment and Collection for Total Iron Quantitation and Proteomic Analyses

To overcome the fact that cell culture conditions are often iron limiting, and to more closely mimic in vivo conditions, cells were cultured in media supplemented with 10% Cytiva HyClone™ Iron-Supplemented Calf Serum (Fisher Scientific; Hampton, NH, USA) for at least 24 h prior to experimental treatments. Transfected H1299 cells were treated with 50 µM deferoxamine (DFO) to induce iron deficiency, while control cultures were treated with the DMSO drug vehicle control. After 48 h, the media was removed, and the cells were dissociated from the flask using Cell Stripper (Corning; Corning, NY, USA). Cells were then collected in PBS and centrifuged at 1000 rpm for 5 min at 4 °C. Cell pellets were either stored at −80 °C for quantitation of total iron levels by ICP-MS at the Iron and Heme Core facility at the University of Utah or solubilized in buffered urea-reducing solution (8 M urea, 100 mM Tris, 5 mM TCEP, pH 8.5) before protein quantitation by bicinchoninic acid assay and proteomics analyses at the Genomics and Proteomics Center at Oklahoma State University.

### 2.3. Tandem Mass Tag Proteomics

Lysates were adjusted to 80 µg of cell lysate per 50 µL lysis buffer. Proteins were then alkylated with 10 mM iodoacetic acid for 30 min at room temperature. Reactions were diluted 8-fold in 100 mM Tris-HCl, pH 8.0, and digested with 4 µg of trypsin/LysC (Thermo Fisher Scientific; Waltham, MA, USA, Promega catalog V5071) at 37 °C. After overnight incubation, another 2 µg of trypsin/LysC was added, and the reactions were incubated for another 6 h. Reactions were then desalted on C18 spin columns following the manufacture’s recommendations (Thermo Fisher Scientific; Waltham, MA, USA, Pierce catalog 89852). Four percent of the sample eluate was aliquoted for use in peptide quantitation (below), and the rest of the eluate was divided into two even aliquots for storage prior to tandem mass tag (TMT) labeling. After aliquoting, all samples were dried by centrifugation under vacuum and stored at −80 °C. Peptide yield was quantified by fluorometry, following the manufacturer’s recommendations (Thermo Fisher Scientific; Waltham, MA, USA, Pierce catalog 23290). Additionally, two pools of proteins were created to serve as a bridge between different TMT labeling experiments. For this bridging pool, proteins from 7 different cell lines treated with DFO were pooled. To make a second bridging pool, proteins from 7 different untreated control cultures of these same cell lines were pooled. Both lysate pools were digested with trypsin, and the product peptides were desalted and labeled with individual TMT channels as described below.

For TMT labeling, samples were redissolved in 50 mM triethylammonium bicarbonate buffer, pH 8.5, and 35 ug of each peptide sample was labeled with 100 µg of TMT reagent [10]. The coding of individual experiments with each TMT channel is described in Supplementary Table S1. After TMT labeling, reactions were quenched with 0.5% hydroxylamine, and a multiplex mixture was created by mixing 10 µL each of TMT reaction from 8 experimental lysates, 10 µL from each of the bridge reactions, and 200 µL of 0.1% TFA. The multiplex mixture was loaded onto a high-pH-resistant C18 spin column (Thermo Fisher Scientific; Waltham, MA, USA, Pierce catalog 84868), desalted by washing with trimethylamine-buffered 5% acetonitrile, and step-fractionated into 11 fractions using 7.5, 10, 12.5, 15, 17.5, 20, 22.5, 25, 27.5, 30, 35, and 60% acetonitrile buffered with trimethylamine. Each fraction was dried by centrifugation under vacuum and stored at −80 °C.

TMT multiplex fractions were dissolved in chromatography mobile phase A (0.1% aqueous formic acid) for injection onto the LC-MS/MS system. After dissolution, approximately 0.5 µg of the 7.5% fraction and 0.5 ug of the 27.5% fraction were combined for a single injection onto the LC-MS/MS. Similarly, the 10% and 30% fractions were combined and injected together, as were the 12.5% and 35% fractions. All of the other fractions were injected individually (ca. 1.0 µg injections each). Peptides were separated using a 75-µm × 50-cm PepMap C18 column (Thermo Fisher Scientific; Waltham, MA, USA, catalog 164942) plumbed in a vented trap configuration. The column was developed with 80:20:0.1 acetonitrile/water/formic acid as mobile phase B, using a 3-h non-linear gradient of 5–17% B (101 min), 17–26% B (62 min), and 26–34% B (17 min). Eluting peptides were ionized in a Nanospray Flex ion source (Thermo Fisher Scientific; Waltham, MA, USA) by application of 1900 V to a stainless-steel needle at the column terminus.

Peptide ions were analyzed using a synchronous precursor selection MS3 method [11]. For this, intact peptide ions were analyzed in the Orbitrap detector, followed by precursor selection using the quadrupole, MS2 fragmentation in the ion trap, and MS2 fragment ion detection in the ion trap. For each peptide ion, this “sequencing” MS2 scan was followed by a second quadrupole selection and CID fragmentation event, from which the 10 most abundant fragments were retained in the ion trap, then fragmented by high-energy collision in the ion routing multipole, and then detected in the Orbitrap mass analyzer. The specific instrument settings are provided in Supplementary Table S2.

Instrument RAW files were searched using the reporter ion MS3 module of MaxQuant v2.0.3.0, specifying 10-plex TMT labeling and TMT lot-specific correction factors. A reporter mass tolerance of 0.003 was specified, and TMT signals were measured as ratios to both reference channels (the pooled bridge samples described above). An isobaric weight adjustment was not used (the Isobaric weight exponent value was set to zero). Carbamidomethylation of Cys was specified as a constant modification, while variable modifications for identification and quantification included oxidation of Met, acetylation of the protein N-terminus, Gln cyclization to pyro-glutamate, and deamidation of Asn and Gln. Two missed trypsin cleavages were tolerated. MS/MS data were searched against a database of 78,139 human proteins downloaded from Uniprot on 24 September 2021, using the default MaxQuant search parameters. Match between runs was used to transfer peptide spectrum matches between RAW files, and second peptides were not searched.

### 2.4. Bioinformatics

The results of the MaxQuant search were analyzed within the Perseus software application, version 1.6.15.0 (Max Planc Institute of Biochemistry, Planegg, Germany) [12]. Decoy proteins and probable contaminants were filtered from the protein dataset. The normalized protein ratios for each individual experiment (vs. the two sample pool reference channels), which were calculated by MaxQuant, were doubled to account for the use of two reporter channels. Ratios were further transformed by log2 transformation, and then normalized to the mean ratio value for each individual experiment. To validate the significance of differences between treatments or cell lines, the significance analysis of microarrays algorithm [13] was utilized within Perseus (“volcano plot tests”), specifying 250 randomizations, and with FDR and s0 values set at 0.05 and 0.10, respectively.

Official gene IDs from the dataset were uploaded to the gene renaming tool within the Database for Annotation, Visualization, and Integrated Discovery (DAVID) tool from the Laboratory for Human Retrovirology and Immunoinformatics [14]. The resulting list of official gene symbols and their corresponding ensemble gene IDs were then added to a separate excel file. Those that were not autogenerated by DAVID were manually added to the renaming array using information from Gene Cards—the human gene database (Weizmann Institute of Science and Life Map Sciences) [15]. Excel’s VLOOKUP tool was used to create a new column containing the corresponding ensemble gene ID for each protein. These ensemble gene IDs were then uploaded to DAVID’s functional annotation tool. From this output, functional annotation and biological process (BP) GO Term analysis results were used for further interpretation using only those proteins detected within our samples as a background gene list.

### 2.5. Enzymatic Assays

Cytosolic and mitochondrial aconitase activities were assessed by measuring the conversion of citrate to isocitrate and normalizing to total protein using an Aconitase Activity Assay kit (MilliporeSigma; Burlington, MA, USA). Glutathione peroxidase (GPX) activity was measured by collecting and resuspending 1 × 10^7^ cells in 60 µL sample buffer (50 mM Tris-HCl, pH 7.5, 5 mM EDTA, and 1 mM DTT) before centrifugation at 10,000× *g* for 15 min at 4 °C. Fifty microliters of the supernatant was used to measure GPX activity via a coupled reaction with glutathione reductase using a Glutathione Peroxidase Assay Kit (Cayman Chemical Company; Ann Arbor, MI, USA), according to kit instructions. The remaining 10 µL of sample was analyzed for total protein content using a Pierce BCA Protein Assay Kit (Thermo Fisher Scientific; Waltham, MA, USA) for normalization.

### 2.6. RNA Isolation and Real-Time qPCR

Total RNA was isolated from confluent 6-well plates of cells expressing endogenous WT and mutant TP53 using TRIzol reagent (Thermo Fisher Scientific; Waltham, MA, USA), according to the manufacturer’s instructions. RNA integrity was confirmed by agarose gel electrophoresis. RNA purity was established by spectrophotometry using a Nanodrop-One (Thermo Fisher Scientific; Waltham, MA, USA) before reverse transcription with SuperScript II (Invitrogen; Waltham, MA, USA). The relative mRNA abundance of thioredoxin reductase 1 (TXNRD1) was determined by qPCR using SYBR green chemistry on a Bio-Rad CFX Opus 384 Real Time PCR system (Bio-Rad; Hercules, CA, USA) and normalized relative to peptidylprolyl isomerase B (PPIB) PPIB abundance using the 2^−∆∆Ct^ method. TXNRD1 and PPIB primer sequences were obtained from previously published sources [9,16].

### 2.7. Viability Assays

To assess resistance to lipid excess, a working stock of palmitic acid solution was prepared as previously described [17]. Briefly, palmitic acid (Millipore-Sigma; Burlington, MA, USA) was diluted to 100 mM in 0.1 M NaOH in a heat bath set to 70 °C. Simultaneously, a 10% weight per volume BSA solution was prepared and heated to 55 °C in a heat block. The fatty acid solution was added dropwise into the BSA solution while in the heat block, until the working concentration was achieved. Solutions were immediately vortexed for 10 s followed by a 10-min incubation at 55 °C. Post incubation, solutions were allowed to reach room temperature prior to being stored at −20 °C. Before use, the FFA solutions were heated to 55 °C and cooled to room temperature. SW48, AU565, and NCI-H510 cell lines were plated at equal densities in a 96-well cell culture plate and treated with either a vehicle control, 250 µM palmitic acid, or 500 µM palmitic acid. After 24 h, cell viability was analyzed using a CCK-8 kit (Selleckchem, Houston, TX, USA) according to the manufacturer’s instructions.

### 2.8. Statistical Analyses

Differences between wild-type and mutant TP53-expressing cell lines were assessed by one-way ANOVA. Protein correlations were determined using linear regression analyses. Differences between cell lines with and without DFO treatment were analyzed using a two-factor mixed design ANOVA, followed by B-H correction to adjust for multiple pairwise comparisons. Student’s *t*-tests were used to determine differences relative to controls within a given cell type. Statistical tests of the proteomics datasets were performed using Perseus. All other statistical tests were performed using SAS Analytics Software version 9.04 (SAS Institute, Inc., Cary, NC, USA). Differences were considered statistically significant at the 95% confidence level (alpha = 0.05). Statistical parameters were calculated for all variables and included mean ± SEM. In this instance, SEM was selected due to the lower biologic variability anticipated with cell line use. Thus, a measure of the certainty and precision around the estimate of the mean was more appropriate than a measure of the dispersion of the samples [18].

## 3. Results

### 3.1. In-Depth Acquisition of the Mutant TP53 Proteome

**Figure 1:**
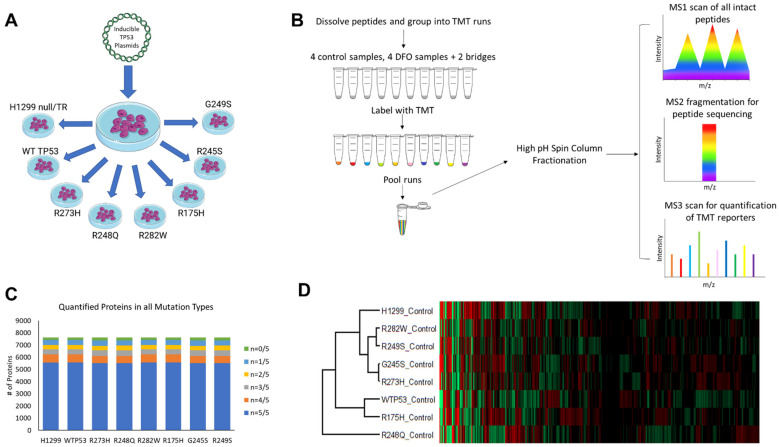
In-depth characterization of the mutant TP53 proteomes. (**A**) Eight isogenic cell lines were generated by transfecting TP53 null H1299 cells with inducible expression plasmids containing either WT TP53 or one of the six indicated TP53 mutation types. (**B**) Cells were treated with ± DFO for 48 h before total protein was collected and lysates were digested with trypsin, labeled with isobaric TMT tags, and pooled, followed by high-pH reversed phase fractionation and analysis by LC-MS/MS. (**C**) Quantified proteome depth including the number of proteins within each biologic replicate. (**D**) Hierarchal cluster analysis of distinct TP53 proteomes in untreated cells.

To obtain a broad and representative overview of the mutant TP53 proteome, we measured the impact of inducing the expression of the most common TP53 mutations in human cancer in great depth using an advanced TMT-based proteomics workflow. We started with TP53 null H1299 cells, and then transfected these cells with a plasmid containing wild-type TP53, or one of the six most commonly expressed TP53 mutation types. These mutation types were selected because they represent nearly 25% of all the TP53 mutations expressed in human cancers. Next, to determine how mutant TP53-expressing cells maintain iron homeostasis in response to reduced iron availability, we treated each of these cell lines with the potent iron chelator, deferoxamine (DFO). From a total of 80 samples (5 control samples and 5 DFO samples from each cell type), proteins were isolated by boiling and sonication in buffered guanidine, quantified by bicinchoninic acid assay, trypsinolyzed, and subjected to orthogonal chromatography [19,20]. Our aim was to compare changes in protein expression among the cell lines and treatments. Thus, each sample was labeled with tandem mass tags, pooled, and fractionated, and then each fraction was analyzed by liquid chromatography-tandem mass spectrometry (LC-MS/MS). Peptide ions were analyzed using a “Top-Speed” data-dependent MS3 method. Peptides were identified and protein ratios were measured by database searching using MaxQuant v2.0.3.0. Applying a strict peptide and protein false discovery rates of 1%, we identified 105,488 total peptides, which includes 85,165 unique peptides that were assigned to 7625 protein groups. We quantified on the basis of uniques and razors, using protein grouping and principles of parsimony. Our workflow quantified 5165 proteins that were present in each of the 80 samples, 5181 proteins in each of the untreated control cells, and 5171 proteins in each of the DFO-treated cell lines (Figure 1).

### 3.2. Iron Chelation Elicits Widespread Changes in Total Protein Expression in Wild-Type and Mutant TP53-Expressing Cells

**Figure 2:**
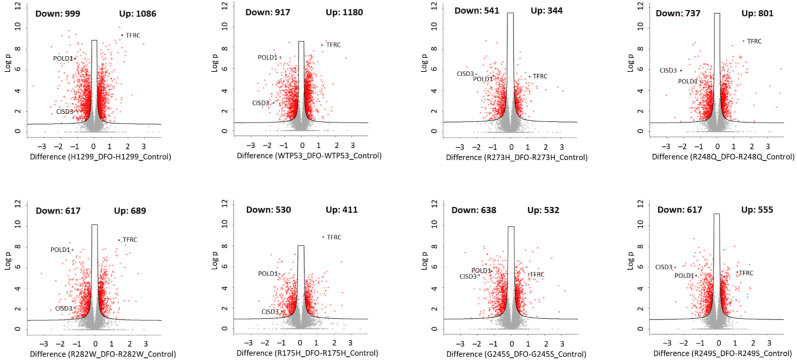
The influence of iron chelation on the mutant TP53 proteome. Volcano plots summarizing significantly (*q* < 0.05) downregulated and upregulated proteins in TP53 null H1299 cells, or H1299 cells expressing WT TP53, or a representative “hotspot” TP53 mutation type treated ± DFO (50 µM, 48 h).

To identify candidate pathways impacting iron metabolism and iron homeostasis downstream of wild-type and mutant TP53, we performed quantitative proteomics analyses on the LS-MS/MS data from each DFO-treated TP53 null, wild-type TP53, and mutant TP53 cell line compared to their respective untreated controls. As expected, all cell types exhibited changes in gene expression consistent with the induction of iron deficiency. DFO induced strong perturbations in the proteomes expressed in all cell types (Figure 2). The expression of the iron uptake protein, transferrin receptor (TFRC) was amongst the most highly upregulated proteins and was increased 2–3-fold in all cell types, whereas iron-containing proteins such as CDGSH iron sulfur domain 3 (CISD3) and DNA polymerase delta 1 (POLD1) were amongst the most downregulated. In both the TP53 null and wild-type TP53-expressing cells, DFO treatment resulted in the upregulation of over 1000 other proteins and downregulation of more than 900 proteins (Figure 2). Thus, the vast majority of DFO-induced changes in protein expression are largely TP53 independent. In contrast, DFO treatment of mutant TP53-expressing cells impacted a significantly lower total number of proteins than that observed in TP53 null or wild-type TP53-expressing cells (Figure 2). These findings are consistent with the notion that mutant TP53 expression significantly influences the way cells respond to changes in iron availability.

**Figure 3:**
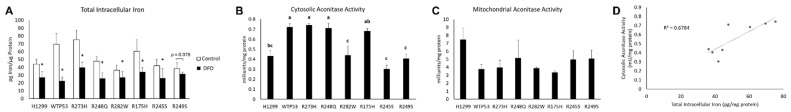
Mutant TP53 expression influences intracellular iron availability and the activity on iron-containing enzymes. (**A**) Total intracellular iron in cells expressing distinct TP53 mutation types under control conditions and following treatment with 50 µM DFO for 48 h. (**B**) Cytosolic (**C**) and mitochondrial aconitase activity in cells expressing distinct TP53 subtypes. (**D**) Cytosolic aconitase activity is directly correlated to levels of intracellular iron under control conditions. * Denotes statistical difference from respective control, *p* < 0.05. Superscripts (a, b, c) denote statistical difference between TP53 subtypes. Subtypes with the same superscripts are not statistically different. Error bars indicate SEM.

We hypothesized that the differences in DFO-mediated changes in protein expression were due to differences in total cellular iron content between cells expressing distinct TP53 mutation types. Indeed, induction of WT and mutant TP53 expression alone was sufficient to alter total intracellular iron levels, and reductions in total intracellular iron levels in response to DFO treatment were smaller in mutant TP53-expressing cells (Figure 3A). However, neither basal total cellular iron levels, nor changes in total iron concentrations in response to DFO treatments correlated with the total number of proteins whose expression was perturbed (Supplemental Figure S1). Importantly, despite varying degrees of responsiveness to iron chelation, the significant increase in TFRC expression combined with significantly decreased total iron levels in all cell types, indicate that the treatment was effective.

Protein expression levels are not always reflective of protein activity levels. Thus, we also measured TP53-dependent differences in the activity of two iron-containing enzymes, mitochondrial and cytosolic aconitase (Figure 3B,C). Modest but statistically significant differences in cytosolic aconitase activity were observed between cells exhibiting distinct TP53 mutation types, whereas mitochondrial aconitase activity was unaffected. Intriguingly, differences in the basal levels of cytosolic aconitase activity directly correlated with differences in basal iron levels (Figure 3D). These findings indicate that TP53-mediated differences in intracellular iron significantly influence the activity of iron-containing proteins, which likely contributes to differences in sensitivity to reduced iron availability.

**Figure 4:**
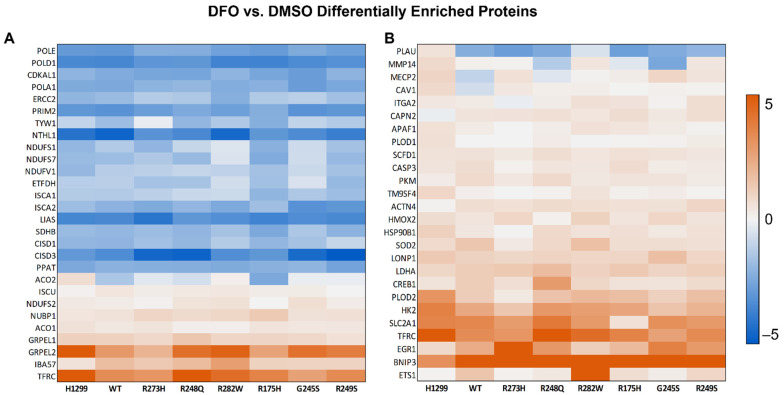
Cells expressing distinct TP53 mutation types exhibit similar protein expression pattern changes in response to iron restriction. (**A**) Differential protein expression summary for proteins with annotated functions in Fe-S cluster biogenesis and (**B**) the cellular response to hypoxia from cells described in Figure 1. Legend scale is the ratio of the log_2_ fold-change (DFO/DMSO).

We then utilized our proteomics dataset to develop specific hypotheses concerning the regulation of iron metabolism in cells expressing distinct TP53 mutation types. To develop these hypotheses, we utilized The Database for Annotation, Visualization, and Integrated Discovery (DAVID) bioinformatics platform. Predictable pathways that would be expected to be influenced by iron chelation, such as the cellular response to hypoxia and iron-sulfur (Fe-S) cluster biogenesis, were amongst the most downregulated and upregulated pathways, respectively, regardless of TP53 status (Figure 4). As shown in Figure 4, the changes in protein expression patterns within these up- and downregulated pathways were highly similar amongst the various TP53 mutation types. Overall, our bioinformatics analyses indicated that the majority of biologic processes impacted by iron deficiency were similarly influenced, despite TP53 mutation type.

### 3.3. Distinct Mutant TP53 Protein Expression Patterns Influence Antioxidant and Lipid Handling Capacity

**Figure 5:**
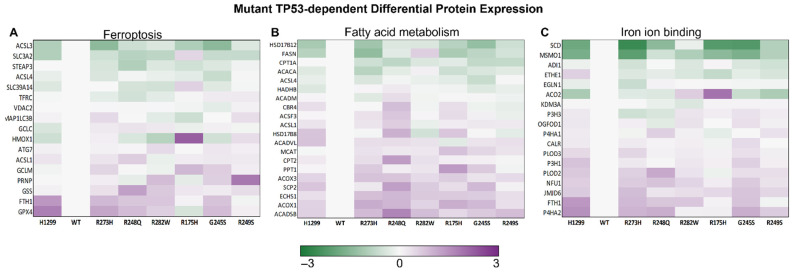
Mutant TP53 expression influences basal levels of protein expression that can directly and indirectly influence iron homeostasis. (**A**) Differential protein expression summary for proteins with annotated functions in ferroptosis, (**B**) fatty acid metabolism, and (**C**) iron ion binding from control cell lines described in Figure 1. Legend scale is the ratio of the log_2_ fold-change (wild-type TP53-expressing cells (WT) and cells expressing the indicated TP53 mutant).

In relation to the bioinformatics analyses, we asked whether metabolic pathways influenced by the induction of TP53 alone might influence the degree to which cells respond to iron chelation. To answer this question, differences in protein expression in untreated control WT and mutant TP53 protein samples were analyzed by one-way ANOVA, and these were then used to identify biological themes. The KEGG pathways and GO terms most significantly impacted by TP53 mutation status alone were fatty acid metabolism, ferroptosis, and iron ion binding (Figure 5). The differences in protein expression patterns between distinct mutant TP53 subtypes within these pathways were further examined to elucidate the potential mechanisms that could contribute to differences in iron availability and ferroptosis sensitivity between the individual mutant subtypes.

**Figure 6:**
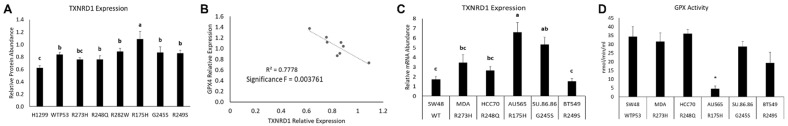
TXNRD1 protein levels in mutant TP53-expressing cells correlates with GPX abundance and activity. (**A**) Relative abundance of TXNRD1 protein levels in TP53 null H1299 cells (H1299), or H1299 cells expressing tetracycline inducible plasmids containing either wild-type TP53 (WTP53) or one of the designated TP53 mutation types was measured by quantitative LC MS/MS following 48 h treatment with 10 µM tetracycline. (**B**) TXNRD1 protein abundance is inversely correlated to GPX protein abundance in H1299 TP53 null, WTP53, and TP53 mutant-expressing cell lines. (**C**) TXNRD1 mRNA expression was measured by qPCR in human cancer cell lines expressing endogenous WT TP53, or one of the TP53 mutation types indicated. (**D**) GPX activity in cells expressing endogenous WT and mutant TP53 was measured indirectly via a coupled reaction with glutathione reductase and normalized to total protein. * Denotes statistical difference from respective control, * *p* < 0.05. Superscripts (a, b, c) denote statistical difference between TP53 subtypes. Subtypes with the same superscripts are not statistically different. Error bars indicated SEM.

One of the hallmarks of ferroptosis is loss of lipid peroxide repair, which leads to the toxic accumulation of lipid reactive oxygen species (ROS) [1]. Thus, we were intrigued by the finding that glutathione peroxidase 4 (GPX4) expression was modestly upregulated in most mutant TP53-expressing cells, except for the R175H mutants (Figure 5A). GPX4 is the central antioxidant enzyme for protection against lipid peroxidation during ferroptosis, and its loss is sufficient to induce ferroptosis in many cell types [21]. Selenoprotein thioredoxin reductase 1 (TXNRD1) has been proposed to cooperate with GPX4 to protect against ferroptosis, but TXNRD1 protein expression has also been shown to negatively affect GPX4 expression in some contexts [22]. Similarly, we observed that TXNRD1 expression was highest in the R175H TP53-expressing mutants (Figure 6A), and that TXNRD1 expression was inversely correlated with GPX4 expression in all of the analyzed TP53 subtypes (Figure 6B).

To determine whether this was a cell-line specific phenomenon, we then analyzed TXNRD1 mRNA expression in a panel of cell lines expressing endogenous versions of analogous wild-type and mutant TP53 variants. In agreement with the results of our isogenic cell lines, AU565 cells, which express an endogenous R175H mutant TP53, had the highest level of TXNRD1 mRNA expression (Figure 6C). To assess the functional consequences of differential TXNRD1 between TP53 subtypes, we measured GPX activity in the same panel of endogenous TP53-expressing cell lines and found that GPX activity was lowest in the R175H mutant expressing AU565 cell line (Figure 6D).

**Figure 7:**
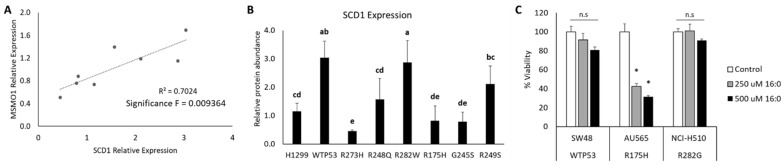
SCD1 protein expression correlates with MSMO1 abundance and improved lipid handling in cells expressing distinct TP53 subtypes. Relative abundance of MSMO1 and SCD1 protein levels in TP53 null H1299 cells (H1299), or H1299 cells expressing tetracycline inducible plasmids containing either wild-type TP53 (WTP53) or one of the designated TP53 mutation types, were measured by quantitative LC MS/MS following 48 h treatment with 10 µM tetracycline. (**A**) MSMO1 and SCD1 protein expression correlation was determined by linear regression analysis. (**B**) Bar graph representing the quantitative differences in SCD1 expression between TP53 mutation types. (**C**) Differences in cell viability in cells expressing endogenous WT TP53 (SW48), an R282G TP53 mutation (NCI-H510), of an R175H mutation (AU565) following treatment with a vehicle control (Control), 250 µM, or 500 µM palmitic acid (16:0) for 24 h. Superscripts (a,b,c) denote statistical difference between TP53 subtypes. Subtypes with the same superscripts are not statistically different. * Denotes statistical difference from respective control, * *p* < 0.05, n.s. = not signifinct. Error bars indicated SEM.

Another hallmark of ferroptosis is the oxidation of polyunsaturated fatty acid (PUFA)-containing phospholipids [1], whereas monounsaturated fatty acids (MUFAs) can promote a ferroptosis resistant state [21]. Thus, stearoyl-CoA desaturase 1 (SCD1) stood out because of its essential role in saturated fatty acid desaturation and its iron binding properties (Figure 5C) [22,23]. Previous research has shown that, in cancer, SCD1 protein expression positively correlates with methylsterol monooxygenase 1 (MSMO1) expression and can confer ferroptosis resistance [22]. We also observed a statistically significant positive correlation between SCD1 and MSMO1 protein expression (Figure 7A). The highest levels of SCD1 protein expression were found in the WT TP53 and R282W TP53-expressing mutants (Figure 7B). These findings expand upon our previous work demonstrating that cells expressing these two TP53 subtypes are less sensitive to ferroptosis compared to TP53 null and other mutant TP53-expressing cell lines [8].

To examine the functional impact of elevated SCD1 expression on handling saturated fatty acid excess, we treated cells expressing endogenous WT TP53 (SW48), an R282G TP53 mutation (NCI-H510), or an R175H mutation (AU565, as a positive control) with 250 and 500 µM palmitic acid (16:0). The NCI-510 cell line was selected because a commercially available cell line with an R282W mutation was not available, and loss of the positively charged arginine at the same critical point in the DNA binding domain is expected to elicit the same phenotypic outcome. Consistent with the predictions from our model, the WT TP53 (SW48) and R282G mutant (NCI-H510)-expressing cell lines were significantly less sensitive to saturated fatty acid-induced lipotoxicity than the AU565 cells (Figure 7C).

## 4. Discussion

Despite the frequency with which TP53 mutations occur, the generic term “mutant TP53” is not sufficient to describe the vast spectra by which distinct TP53 mutation types influence tumor cell phenotypes and metabolic functions. To examine these phenotypes further, we generated isogenic cell lines expressing the six most common TP53 mutation types in human cancers [24]. Importantly, these six mutation types also represent the most common examples of “DNA contact” (R273H, R248Q, and R282W) and “conformational” (R175H, G245S, and R249S) mutants. DNA contact mutants express missense mutations in amino acid residues that make direct contact with target DNA sequences, whereas conformational mutations disarrange the structure of the P53 protein [25]. However, herein we show that even these two categories are too broad to infer how a given TP53 mutation type will influence basal cell protein expression as hierarchal cluster analysis failed to group cells expressing these two mutation subtypes together. Cells expressing mutants categorized within these two broad subtypes also displayed divergent sensitives to iron chelation by DFO treatment. As our results indicated that differences in proteome sensitivities were not due to differences in intracellular iron content before or after iron treatment, we utilized a bioinformatics approach to elucidate the physiology behind these disparate responses.

Differences in basal intracellular iron levels and iron-containing enzymatic activity following the induction of WT and mutant TP53 expression have previously been attributed to WT TP53-dependent regulation of iron regulatory proteins and Fe-S cluster biogenesis [9,26,27]. Thus, we hypothesized that TP53-dependent differences in iron regulatory and Fe-S-containing proteins would influence the response to reduced iron availability. However, neither differences in Fe-S cluster biogenesis related proteins, nor Fe-S containing protein activity explained the divergent responses to DFO treatment. Given the indispensable nature of Fe-S clusters to cellular vitality [28], it makes physiologic sense that the functions of the proteins involved in these pathways are preserved. The activation of proteins involved in the cellular response to hypoxia in all cell types [29,30], which would be predicted in response to impaired oxygen delivery as a result of iron chelation, also indicates a highly preserved response to iron starvation to sustain essential life-preserving processes.

Next, we explored how TP53-dependent changes in protein expression independently of DFO treatment might provide insights into the observed differences in sensitivities to iron availability. We were particularly interested in identifying alterations in pathways and proteins that could be used to exploit the toxic nature of iron [31] and the reduced capacity for mutant TP53 to protect cells from iron-mediated, ferroptotic cell death [3,4,8]. Besides iron, ferroptosis is also dependent on the oxidation of PUFA-containing phospholipids and the loss of lipid peroxide repair capacity; thus, mutant TP53-dependent alterations in SCD1 and GPX4 protein expression stood out and were scrutinized further.

SCD1 catalyzes the desaturation of saturated fatty acids to their MUFA counterparts, such as palmitic acid (16:0) to palmitoleic acid [32]. This property, in addition to the upregulation of other genes involved in fatty acid metabolism, enables SCD1 to protect cancer cells from ferroptotic cell death [22,32]. The discovery that SCD1 expression is highest in isogenic cell lines transfected with exogenous WT TP53 and R282W mutant TP53 provides clarity to our previous work showing that these two cell lines are less sensitive to ferroptosis than the other mutant TP53 subtypes [8]. In contrast, induction of WT TP53 has also been shown to represses SCD1 expression [33]. However, in those experiments, WT TP53 was overexpressed in a cell line harboring an endogenous TP53 mutation to levels that were sufficient to induce apoptosis, and the levels of SCD1 expression under control conditions were not reported, which could explain these discrepancies. To expand upon our proteomics results, we investigated how increased SCD1 expression influences the capacity to handle saturated fatty acid excess. Adding credence to our findings, we observed that cells expressing endogenous WT TP53 and a comparable R282G TP53 mutation are indeed less sensitive to saturated fatty acid-induced lipotoxicity.

Another enzyme that is critical for protection against lipotoxicity is the selenoenzyme GPX4. Inactivation of GPX4 leads to the toxic accumulation of lipid ROS, which can drive ferroptosis [34]. In pancreatic cancer cells, loss of TXNRD1 results in increased abundance of GPX4 protein, and confers protection from ferroptosis [35]. Likewise, we also found that TXNRD1 protein expression inversely correlated to GPX4 protein expression, as well as GPX activity. Higher TXNRD1 protein expression in mutant TP53-expressing cells compared to the TP53 null cells is also in agreement with previous findings demonstrating that R175H and R273H TP53-expressing mutants are more sensitive to the cytotoxic effects of the TXNRD1 inhibitor auranofin [36]. However, cell death appeared to result from the activation of both ferroptotic and apoptotic pathways, and the role of GPX4 in the response to TXNRD1 inhibition was not fully investigated.

Intriguingly though, GPX4 inhibition with the ferroptosis inducer RSL3 has been shown to lead to a time- and dose-dependent increase in the expression of the cytoprotective enzyme heme oxygenase 1 (HMOX1) [37]. Similarly, we found that R175H TP53 mutants, which expressed the lowest levels of GPX4 protein, also expressed the highest levels of the HMOX1 protein. As loss of HMOX1 protein expression can augment ferroptotic cell death following treatment with GPX4 inhibitors, these findings have therapeutic implications [37]. Future studies should investigate the potential for HMOX1 inhibitors to synergistically improve the efficacy of ferroptosis induction in cancer cells expressing R175H TP53 mutations.

## 5. Conclusions

Mutations in TP53 do not only result in loss of tumor suppressive functions but can also lead to the alterations of the transcriptional programs that actually drive tumorigenesis. Thus, the discovery that mutations in TP53 may also increase ferroptosis sensitivity presented an exciting avenue for effectively targeting mutant TP53, but the mechanisms driving this increased sensitivity vary depending upon TP53 mutation type [3,4,5,6]. Given the essentiality of iron accumulation to the effectiveness of ferroptosis activation, we explored how expression of the most common TP53 mutation types influences cellular iron homeostasis. The findings in this study indicate that differential responses to iron availability in cells with distinct TP53 mutation types are not due to differences in how cells directly handle iron. Rather, differences in antioxidant and lipid handling protein expression profiles likely alter the setpoint at which cells expressing distinct TP53 subtypes maintain iron homeostasis. These findings expose the distinct weaknesses of some of the most common TP53 mutation subtypes and provide important insights for the focus of future investigations aimed at developing better therapeutic regimens for targeting mutant TP53.

## Data Availability

All quantitative proteomics expression data are provided in Supplementary file Table S3.

## Supplementary Materials

The following supporting information can be downloaded at: https://www.mdpi.com/article/10.3390/cells11132064/s1, Table S1: TMT_KeyTable; Table S2: MS_Programming; Table S3: Raw proteomics expression data file; Figure S1: Total iron changes.
