# Supplementary figures and images for: Differences in Antioxidant and Lipid Handling Protein Expression Influence How Cells Expressing Distinct Mutant TP53 Subtypes Maintain Iron Homeostasis
Source: Cells. 2022 Jun 29;11(13):2064. doi: 10.3390/cells11132064 (PMC9265551; doi:10.3390/cells11132064)

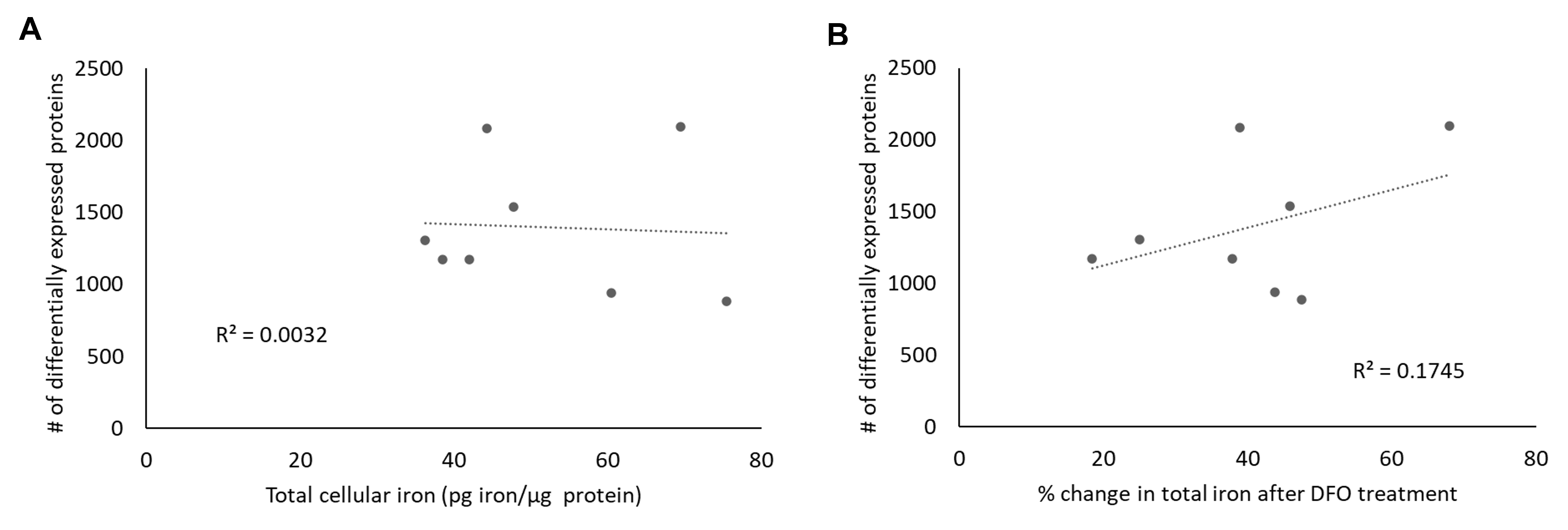

Supplement: Supplementary file 1 [file cells-11-02064-s001.zip › Supplemental Figure S1.png]
